# Biopsychosocial Associates of Psychological Distress and Post-Traumatic Growth among Canadian Cancer Patients during the COVID-19 Pandemic

**DOI:** 10.3390/curroncol31090395

**Published:** 2024-09-10

**Authors:** Karen M. Zhang, Som D. Mukherjee, Gregory Pond, Michelle I. Roque, Ralph M. Meyer, Jonathan Sussman, Peter M. Ellis, Denise Bryant-Lukosius

**Affiliations:** 1 Juravinski Cancer Centre, Hamilton Health Sciences, Hamilton, ON L8N 3Z5, Canada; 2 Department of Psychiatry and Behavioural Neurosciences, McMaster University, Hamilton, ON L8S 4K1, Canada; 3 Department of Oncology, McMaster University, Hamilton, ON L8S 4K1, Canada; 4 Escarpment Cancer Research Institute, Hamilton, ON L8V 5C2, Canada; 5 School of Interdisciplinary Science, McMaster University, Hamilton, ON L8S 4K1, Canada; 6 School of Nursing, McMaster University, Hamilton, ON L8S 4K1, Canada

**Keywords:** anxiety, biopsychosocial models, cancer, COVID-19, depression, emotional distress, oncology, health social determinants

## Abstract

Objective: Understanding both the positive and negative psychological outcomes among cancer patients during the pandemic is critical for planning post-pandemic cancer care. This study (1) examined levels of psychological distress and post-traumatic growth (PTG) among Canadian cancer patients during the COVID-19 pandemic and (2) explored variables that were associated with psychological distress and PTG during the pandemic using a biopsychosocial framework. Method: A cross-section survey was undertaken of patients receiving ongoing care at a regional cancer centre in Ontario, Canada, between February and December 2021. Self-reported questionnaires assessing sociodemographic information, social difficulties, psychological distress (depression, anxiety fear of recurrence, and emotional distress), PTG, illness perceptions, and behavioural responses to the pandemic were administered. Disease-related information was extracted from patient health records. Results: Prevalences of moderate to severe levels of depression, anxiety, fear of recurrence and emotional distress were reported by 26.0%, 21.2%, 44.2%, and 50.0% of the sample (*N* = 104), respectively. Approximately 43% of the sample reported experiencing high PTG, and these positive experiences were not associated with levels of distress. Social factors, including social difficulties, being female, lower education, and unemployment status were prominent associative factors of patient distress. Perceptions of the pandemic as threatening, adopting more health safety behaviours, and not being on active treatment also increased patient likelihood to experience severe psychological distress. Younger age and adopting more health safety behaviours increased the likelihood of experiencing high PTG. The discriminatory power of the predictive models was strong, with a C-statistic > 0.80. Conclusions: Examining both the positive and negative psychological patient outcomes during the pandemic has highlighted the complex range of coping responses. Interventions that adopt a multi-pronged approach to screen and address social distress, as well as to leverage health safety behaviours, may improve the adjustments in the pandemic aftermath.

## 1. Introduction

Emerging evidence suggests that patients with cancer experienced higher levels of psychological distress during the pandemic than the general population [1,2,3,4,5]. Psychological distress among people facing cancer can encompass a number of presenting concerns including low mood, anxiety, fear of recurrence (FCR), and emotional distress [1]. The severity in mental health symptoms has been alarming, with the prevalence of depression and anxiety among patients during this period ranging from 16.7 to 35.0% and 17.7 to 44.4%, respectively [6,7]. This is substantially higher than the pre-pandemic prevalence of 12.9% and 19.0% for depression and anxiety among cancer patients, respectively [7]. Fear of recurrence, which refers to worry about cancer returning or progressing, has been amplified during the pandemic and was estimated to be experienced by 52.8–95.0% of patients [8,9].

On the other hand, it is also possible for patients to experience positive personal changes in response to their experience with the pandemic [10,11]. Post-traumatic growth (PTG) refers to when an individual develops greater appreciation, deeper insights, or stronger bond with others following adverse or traumatic life events [12]. It has been theorized that PTG occurs when an individual is able to make new meaning from their psychological suffering as a result of a distressing experience [13]. Past studies have found that many cancer patients report experiencing PTG after their diagnosis, with higher growth seen amongst individuals who are younger in age, having more perceived social support and having higher education [14,15]. The extent to which cancer patients report PTG as a consequence of the pandemic has not been widely studied. It is also lesser-known what factors are associated with individuals who are more likely to report positive transformative change compared to those who report high distress from the pandemic.

According to the biopsychosocial model, psychological outcome is affected by multiple domains that include biological, psychological, and social dimensions [16]. Psychological factors, which include behaviours and cognitive appraisals, have been shown to contribute to cancer patients’ susceptibility to poor mental health outcomes during the pandemic. Specifically, behaviours used to mitigate the risk of COVID, such as social isolation, have been associated with higher psychological distress among patients [5,17,18]. It has also been reported that the extent to which cancer patients appraise COVID-19 as threatening, a construct referred to as illness perceptions [19,20], has been linked to higher levels of depression and perceived stress during the pandemic [20,21]. Self-compassion and psychological flexibility are cognitive variables among cancer patients that are associated with higher levels of PTG during the pandemic. There are limited data, however, on whether the likelihood of developing psychological distress and/or PTG derive from similar cognitive variables among cancer patients.

Social difficulties or social distress is a term used to describe challenges with tasks related to cancer care, and it includes the domains of money matters, relationship with self and others, and everyday tasks. Several studies have reported on the socioeconomic sequalae of COVID-19 on patients with cancer that include struggles with transportation and medical costs [22,23]. Emerging data suggest that sociodemographic characteristics influence how the pandemic affects patients, with individuals who are younger, Black, female, and unemployed reporting higher distress [18,24]. To date, no research has examined how social difficulties may impact the development of PTG among cancer patients during the pandemic. It is important to understand social factors that can hinder or promote patient adjustment to stressful events in order to develop adequate interventions.

With regard to biological factors, studies have reported mixed findings as to whether disease and medical variables contribute to psychological outcomes during the pandemic. While some studies have noted that pre-existing medical conditions, disease site, and advanced cancer stage were associated with higher levels of FCR and anxiety [25,26], other studies have found no association between disease factors and psychological outcomes [5,9,27]. The extent to which biomedical factors impact a cancer patient’s experience of PTG during the pandemic has not been examined.

Research thus far has mostly examined psychological distress and PTG in isolation, and the relationship between the two constructs are unclear. It is also not well understood which biopsychosocial factors influence patients’ likelihood to experience PTG, depression, anxiety, FCR, and/or distress during the pandemic. A biopsychosocial approach that concurrently considers the correlates of both positive and negative psychological outcomes is critically needed to elucidate salient drivers of psychological outcome and steer the direction of future interventions. As such, the objectives of the present study were to (1) explore levels of psychological distress and PTG among Canadian cancer patients during the COVID-19 pandemic and (2) examine variables that are associated with psychological distress and PTG during the pandemic using a biopsychosocial framework.

## 2. Methods

### 2.1. Participants and Procedures

The present study was an exploratory cross-sectional study using convenience sampling. Potential participants were patients receiving care at a regional cancer centre in Ontario, Canada, between February and December 2021. Eligible patients met the following inclusion criteria: 18 years of age and older, oral and written fluency in English, diagnosis of cancer, and receiving ongoing care at the cancer centre. Methods of recruitment included online posting of study flyers on cancer community websites and social media platforms, distribution of study flyers, and identification of interested potential participants by oncologists. Interested individuals were contacted by the research assistant to screen for eligibility and review the study information. Participants were given the option to complete the survey in paper or electronic form. Consent was implied upon completion of the study questionnaires, which was approximately 15–25 min in length. Three email and/or telephone reminders were sent to non-responders within 6 months after sending the questionnaires. All study protocols were approved by the Hamilton Integrated Research Ethics Board (HiREB #11357).

### 2.2. Measures

#### 2.2.1. Social Factors

Sociodemographic characteristics including age, gender, race/ethnicity, relationship status, number of individuals in the household, household income, level of education, and employment status were assessed using a self-report questionnaire. The validated 21-item Social Difficulties Inventory (SDI-21 [28]) was used to examine the extent to which individuals perceived having difficulties with every day practical problems, such as transit, finances, and social support. Each item was rated on a 4-point scale ranging from “0—no difficulty” to “3—very much difficulty”. A sum score of 10 or greater demonstrated significant social distress. The SDI-21 also had three subscales assessing difficulties with finances (SDI—money), activities of daily living (SDI—everyday), and social relationships (SDI—Others). The SDI and its subscales were validated for use with cancer patients and have shown good internal consistency [28].

#### 2.2.2. Medical Characteristics

A registered oncology nurse reviewed participants’ medical charts to collect clinical data including participants’ primary cancer diagnosis, date of diagnosis, treatment intent (curative, palliative, unknown/control), number of medical comorbidities (i.e., other diagnoses listed on the chart), and treatment status (active, not active). Cases that were deemed ambiguous (9.61% of charts; *n* = 10) were then reviewed by medical and radiation oncologists to confirm participant medical characteristics.

#### 2.2.3. Pandemic-Related Perceptions and Behaviours

COVID-19-related illness perceptions were assessed using an adapted version of the Brief Illness Perception Questionnaire (IPQ [29]). The word “illness” was changed to “COVID-19 pandemic” in the adapted version. The 6-item questionnaire was rated from 0 to 10 to assess the extent to which individuals perceive that they are knowledgeable about COVID-19 and its symptoms, that they will contract COVID, the longevity of the pandemic, degree of COVID impact on their lives, and the degree of control on protecting themselves from the pandemic. The brief IPQ is a widely used measurement that has shown good validity and test–retest reliability [29].

Pandemic-related behavioural responses were measured using 9 items taken from the FluTEST questionnaire (PBQ [29]). The items were adapted by changing the word “flu” to “COVID-19 pandemic”. The survey assessed the extent to which an individual adopted safety behaviours to prevent the risk of contracting COVID, such as sanitizing surfaces and wearing personal protective equipment. Each item was rated on a 4-point scale ranging from “not at all true” to “very true”. Previous studies using a similar questionnaire have demonstrated good reliability and validity [30].

#### 2.2.4. Outcome Variables

(a) *Depressive symptoms* were measured using the Patient Health Questionnaire-9 (PHQ-9 [31]). Each item was rated on a 4-point scale, ranging from “0 = never” to “3—nearly every day,” to determine the frequency to which an individual experienced a depressive symptom. Total scores of 10 or higher indicated moderate to severe levels of depression. The PHQ-9 is a validated and widely used instrument for measuring levels of depression.
(b) *Anxiety symptoms* were measured using the standardized 7-item Generalized Anxiety Disorder (GAD-7 [32]) questionnaire, a validated measure of anxiety-related symptoms among adults. Participants rated the frequency to which they experienced an anxiety symptom using a 4-point scale that ranged from “0 = Never” to “3—nearly every day”. Total scores of 10 or higher indicated moderate to severe levels of anxiety.
(c) *Fear of cancer recurrence* was assessed using the 8-item Cancer Worry Scale (CWS [33]) The CWS identified dysfunctional fear of recurrence among cancer survivors and has demonstrated good construct reliability and validity. Each item was rated on a 4-point scale to determine the frequency to which an individual worried about their cancer progressing or reoccurring. Total scores of 14 or above indicated high levels of fear of recurrence.
(d) *Emotional distress* was measured using the National Comprehensive Cancer Network Distress Thermometer (NCCND Distress [34]). This is an extensively used single-item tool that gauges levels of distress among oncology patients. Participants rated their level of distress on a visual analog ranging from 0 “no distress” to 10 “extreme distress”. Scores above 4 indicated moderate to severe levels of distress.
(e) *Post-traumatic Growth* (PTG) was assessed using the 21-item Post-traumatic Growth Inventory (PTGI [35]). The PGI has been widely used to measure positive outcomes reported by persons who have experienced traumatic events. Participants were asked to rate the degree to which they experienced a positive change due to the pandemic with a 6-point scale, ranging from 0 (I did not experience this change as a result of the pandemic) to 5 (I experienced this change to a very great degree as a result of the pandemic). Responses were summed, with a score of 46 or above indicating high levels of PTG [36].

### 2.3. Analyses

All tests were performed using SPSS version 28.0 (IBM, Armonk, NY, USA, 2021) and SAS. Sample size was determined a priori based on the need for it to be large enough to make reasonable inference about the true estimates of moderate to severe levels of depression, anxiety, FCR, emotional distress, and PTG. This was assumed to occur when the half-width of 95% two-sided conference intervals was less than 0.1. Considering that the rate of psychological distress and PTG in past studies have been less than 0.5, a target sample size of 102 patients was deemed appropriate.

Descriptive statistics were used to summarize sociodemographic, clinical, and outcome data. Univariate analyses were conducted to determine associations between associate factors and outcome data using univariate logistic regression. All associate variables were subsequently entered into a forward stepwise multivariate logistic regression model to determine their relative associations with each outcome variable. The C-statistic was used to measure goodness of fit for the multivariate binary logistic models. C-statistic values over 0.5 indicated that the model was no better at predicting outcomes than random chance, values 0.7 indicated a good model, and values over 0.8 indicated a strong model with high discriminatory power. As there were less than 5% missing values for the survey data, the listwise deletion method was used for all analyses. All analyses were two-tailed and a *p*-value < 0.05 was considered statistically significant.

## 3. Results

### 3.1. Participant Characteristics

A total of 132 patients expressed interest in study participation, and *N* = 104 completed the survey and were included in the analyses. Six individuals declined to participant, seventeen were lost to follow-up, and five people did not meet the study inclusion criteria. Missing data ranged from 0 to 4.8%. The most common participant characteristics were women, married/in common-law relationship, retired, white, obtained a bachelor’s degree or higher education, had a household income less than $60,000 CAD, had curative treatment intent at the time of survey completion, were on active cancer treatment, diagnosed with cancer within 2 years, and were diagnosed with breast cancer (Table 1). On average, participants were 61.58 years old (*SD* = 12.9), had two people living in the household (interquartile range was 2, 3), and had two comorbidities (interquartile range was 1, 4). Table 1 shows the characteristics of the study sample.

### 3.2. Prevalence and Associates of Psychological Distress

#### 3.2.1. Anxiety

The prevalence of high anxiety (GAD-7 ≥ 10) was 21.2%, (*n* = 22; Table 2). The univariate analyses showed that higher levels of social distress, not being on active treatment, and perceiving the pandemic as threatening were associated with severe anxiety (Table S1). After entering all associate factors in the multivariate logistic analysis, only social difficulties and treatment status remained significant associates of GAD-7 scores. Those not on active treatment were 3.37 times (95% CI 1.05 to 10.85) more likely to report moderate to severe levels of anxiety. For every unit increase in SDI, there was an increase of 1.20 (95% CI = 1.10 to 1.32) in the odds of having high anxiety in the multivariate model (see Table 3). The fit of this regression model was strong, with a C-statistic = 0.83.

#### 3.2.2. Depression

Table 2 shows that 26.0% (*n* = 27) of participants reported high levels of depressive symptoms (PHQ-9 ≥ 10). Patients who were single, had a non-white ethnicity, were not on active treatment, had more social difficulties, and had threatening pandemic perceptions were more likely to report severe depression (Table S1). Entering all variables into the multivariate logistic regression showed that those not on active treatment were 3.47 times (95% CI: 1.38 to 8.72) more likely to report moderate to severe levels of depression, and for every unit increase in SDI, there was a 1.26 increase (95% CI: 1.15 to 1.39) in odds of depression (see Table 3). The goodness of fit of this multivariate model was strong, with a C-statistic = 0.88.

#### 3.2.3. Fear of Recurrence

The prevalence of high levels of fear of recurrence (CSW ≥ 14) was 50.0% (*n* = 57). Severe levels of FCR were associated with younger age, being single, employment status, not retired, not being on active treatment, and having greater social difficulties and threatening pandemic perceptions (Table S1). In a forward stepwise logistic regression (Table 3), patients who were working and those who were unemployed/on disability were 0.87 (95% CI: 0.30 to 2.56) and 4.61 (95% CI: 1.23, 17.32) more likely to report high levels of FCR, respectively. For every unit increase in IPQ, there is a 1.13 (95% CI: 1.06 to 1.21) higher likelihood to experience high FCR. The fit of this multivariate logistic model that included employment status and illness perceptions of COVID as associate variables was good, with a C-statistic = 0.79.

#### 3.2.4. Emotional Distress

Within the study sample, 44.2% of participants (*n* = 46) reported elevated levels of emotional distress (NCCND ≥ 4; Table 2). Severe levels of emotional distress were associated with younger age, male gender, employment status, not being on active treatment, having more social difficulties, and having threatening pandemic perceptions (Table S1). In a stepwise multivariate logistic regression that accounted for all associate variables (Table 3), gender, level of education, social distress, and pandemic-related behaviours remained significant predictors of high emotional distress. Females were 6.32 times (95% CI: 1.59 to 25.06) more likely than males to report high levels of emotional distress, and those with a high school or less level of education were 1.09 times (95% CI: 0.30 to 3.99) more likely to than those with university or postgraduate education. For every unit increase in SDI and PBQ, there was a 1.25 (95% CI: 1.14 to 1.38) and 1.23 (95% CI: 1.08 to 1.40) increase in likelihood to experience high emotional distress, respectively. The fit of the logistic model was strong, with a C-statistic = 0.87.

#### 3.2.5. Post-Traumatic Growth

High levels of PTG were reported by 42.7% of the sample (*n* = 44; Table 2). Table 4 shows that PTG levels are positively correlated with FCR (*r* = 0.24, *p* = 0.02) but not with other dimensions of psychological distress. In the univariate analyses, high levels of PTG were associated with younger age, adopting more pandemic health safety behaviours, having curative treatment intent, and viewing the pandemic as threatening (Table S1). In a stepwise multivariate logistic regression, those who were younger in age and adopted more health safety behaviours were more likely to experience high PTG (see Table 3).

## 4. Discussion

To the best of our knowledge, this cross-sectional study was among the few to examine both levels of positive and negative psychological outcomes among Canadian cancer patients during the pandemic. The prevalence of moderate to severe levels of depression (26.0%), anxiety (21.2%), and emotional distress (44.2%) observed in this study were high and within the range obtained with oncology patients from other countries during the pandemic [6,9]. The rate of clinically significant levels of FCR (50.0%) during the pandemic was comparable to results noted in other studies during the same period [8]. Approximately 42.7% of our sample experienced high levels of PTG, which is lower compared to pre-pandemic levels [37]. Taken together, our findings along with others [5,8] illustrate that psychological distress and growth outcomes in the oncology population are worse compared to pre-pandemic data [7], which underscores the profound importance to screen and monitor patients’ mental health needs beyond the pandemic.

A notable finding was that levels of PTG were not related to degree of psychological distress, such that some cancer patients simultaneously reported distress (depression, anxiety, FCR, and distress) and also positive changes during the COVID-19 pandemic. Past studies have suggested that PTG can occur independent of psychological distress because it can serve as an avoidant coping approach to make sense of emotional difficulties during or shortly after the traumatic event [14,38,39]. The term illusory PTG refers to a self-deceptive strategy to re-assure oneself that they are coping better than they actually are [11,40]. It has been found that approximately 17% of individuals in the general population who reported moderate to high levels of PTG during the pandemic did not actually experience true growth. As well, recent evidence suggests that levels of PTG can improve during the course of the pandemic for cancer patients [41]. It is possible that we may observe higher levels of PTG among the oncology population in the aftermath of the pandemic when patients are able to more fully process their experiences.

The second study objective was to examine factors associated with severe psychological distress using a biopsychosocial model. A critical finding was that social factors played a prominent role in patients’ experience of psychological distress. Our findings demonstrate that social difficulties were widespread, with approximately 43% of participants reporting clinically elevated levels of social challenges. Patients who reported having more social difficulties were more likely to report moderate to severe levels of depression, anxiety, emotional distress, and FCR, even after accounting for disease-related and psychological variables. Our results revealed that all facets of social difficulties, such as finances and employment, relationship with self and others, and challenges with everyday tasks (i.e., transportation), were all associated with patient distress. In addition to social distress, being female, not working, having a disability, and having less than high school education were factors linked with a higher likelihood of experiencing more severe emotional distress and fear of recurrence.

On the contrary, social difficulties were not an associate factor of PTG among cancer patients during the pandemic. This supports the notion that the experience of positive changes is driven through a cognitive and spiritual process rather than being a reflection of practical challenges [42]. While it is strongly indicated that the mental health toll of the pandemic disproportionately impacted patients with more socioeconomic and social challenges [8,18,24], these individuals could have experienced positive changes in different ways. For example, the restrictions of the pandemic may have lessened financial and social pressures to entertain and socialize with others. For younger oncology patients, this was perceived as a benefit of the pandemic as it made them feel less behind than their peers [43]. The present study highlights the importance of evaluating both psychological distress and positive psychological outcomes as it shows the complexities to which individuals with cancer responded to and experienced the global pandemic.

Within the biopsychosocial framework, psychological variables had some influence on patient mental health but only regarding FCR and emotional distress. Patients who had more threatening cognitive appraisals about the pandemic were more likely to experience severe FCR. Negative appraisals of COVID-19 vulnerability and FCR both involve an overestimation of risk [7,44]. A dispositional tendency to react negatively to uncertain situations, also known as intolerance of uncertainty [45], may be a factor that underlies the relationship between high FCR and negative illness beliefs.

Interestingly, patients who adopted more health safety behaviours, such as disinfecting surfaces and limiting social contact, were both more likely to experience emotional distress and high PTG. Due to the cross-sectional nature of the study, we were unable to determine the causality of the relationship, but it is possible that individuals who were worried about the health safety of the pandemic relied on preventative behaviours as a coping strategy [46]. The performance of the health safety behaviours may have also led patients to then feel more in control during a time of uncertainty, hence promoting a sense of positive change [47]. Putting it together, our data suggest that distressed cancer patients may have been more likely to perform health safety behaviours, and by doing so, they were more likely to have more positive experiences during the pandemic. This paradoxical relationship aligns with our findings and those of others [38] that PTG and distress can co-exist. While the mechanisms underlying the development of emotional distress and PTG warrant further investigations, our findings suggest that patients can cope through a health crisis when they are given health actions to follow.

With regard to the biological domain of the biopsychosocial model, we generally did not observe linkages between disease-related factors and psychological outcomes, which is consistent with some study findings [5,20,48]. It is possible that our heterogenous sample of cancer patients may have muted the influence of disease-related factors, such as cancer site. Yet, considering the high discriminatory power of our predictive models, we make the argument that disease-related variables may have played a lesser role in influencing patients’ psychological distress during the pandemic.

Our results did indicate, however, that patients who were on active treatment during the pandemic were at least three times less likely to report severe levels of anxiety and depression than those not on active treatment. Other studies have similarly found that patients receiving uninterrupted cancer treatment during the pandemic reported lower levels of psychological distress [23,27]. One explanation could be that patients who were on active treatment had regular face-to-face interactions with their medical providers and thereby received routine emotional support and clinical information during a time of social isolation. Strong therapeutic rapport with medical providers has been linked to greater patient psychological well-being during the pandemic [5,49,50]. An important area for future research is to examine whether the use of virtual care in oncology leverages the same extent of perceived social support for patients during their medical visits.

Several study limitations should be considered. First, the use of a self-selection recruitment method may have biassed participant responses. Second, causal effects between associate and outcome variables cannot be assumed due to the cross-sectional design of the study. As well, the cross-sectional measure of psychological distress at one time point limits our ability to examine changes to patients’ mental status across the evolving phases of the COVID-19 pandemic. The absence of a non-cancer comparison group in this study also limited the interpretation of our results. Third, we cannot elucidate the relationship between psychological outcome and cancer disease site due to the small number of patients with specific cancer types, such as lung and central nervous system. There were also proportionately more breast cancer patients enrolled in our study; thus, our findings are not generalizable to all people facing cancer. Although the present sample was powered for the data analyses, having a larger sample size would allow for subgroup analyses that can detect disease-specific concerns. Future studies should consider more stringent inclusion criteria to enhance the interpretation of the present findings. Fourth, our recruited sample were overrepresented by females (76.7%) and white-Canadians (90.4%). While these data presented a unique gender-specific perspective during the pandemic, using recruitment strategies to obtain a more ethnically and socioeconomically diverse sample would be important to consider for future research. Lastly, there were no instruments measuring COVID-specific illness perceptions and behaviours at the time of study conception. The modification of standardized questionnaires (i.e., IPQ) in this study may have impacted the psychometric properties of the measures.

## 5. Conclusions

This study uniquely used a biopsychosocial lens to examine the associates of positive and negative psychological outcomes among patients with cancer during the pandemic. Our results demonstrated that a significant proportion of Canadian cancer patients experienced severe levels of depression, anxiety, FCR, and emotional distress and lower levels of PTG during the pandemic. Social factors emerged as the strongest determinant of all facets of psychological distress, while perceiving the pandemic as threatening, adopting more health safety behaviours, and not being on active treatment were also associated with negative outcomes. Younger patients and those who followed more health safety behaviours were more likely to report positive experiences due to COVID-19. This study demonstrates that examining both positive and negative psychological outcomes during a health crisis can help elucidate the range of responses and methods of coping during the pandemic. The status of post-pandemic impact on the well-being of individuals facing cancer warrants further study. Using a multi-pronged approach that equips patients with skills to address social concerns and leveraging health actions may help reduce distress and promote growth for people facing cancer in the pandemic aftermath.

## Figures and Tables

**Table 1:** Sociodemographic and medical characteristics of study sample.

Characteristic	Statistic	*n*	Result
Age	Mean (*SD*)	102	61.6 (12.9)
People in Household	Median (IQR)	102	2 (2, 3)
Comorbidities	Median (IQR)	103	2 (1, 4)
	***n* (%)**		
Sex	Female	103	79 (76.7)
Relationship Status	Married/Common-law	102	73 (71.6)
Employment Status	Working	102	34 (33.3)
	Not Working		21 (20.6)
	Retired		47 (46.1)
Ethnicity	White	104	94 (90.4)
Level of Education	High School or Less	101	24 (23.8)
	Some Post-Secondary		37 (36.6)
	University or Postgraduate		40 (39.6)
Household Income (CAD)	<$60,000	104	33 (31.7)
	$60,000 to $99,999		24 (23.1)
	$100,000 or more		26 (25.0)
	No Response		21 (20.2)
Treatment Intent	Curative	104	59 (56.7)
	Palliative		37 (35.6)
	Control/Unknown		8 (7.7)
Active Treatment	Yes	104	78 (75.0)
Disease Site	Breast	104	55 (52.9)
	Genitourinary		21 (20.2)
	Hematology		14 (13.5)
	Other		14 (13.5)
Time Since Diagnosis	Median (IQR) Months	104	23 (10.5, 57.5)
	<1 year		27 (26.0)
	12–23 months		27 (26.0)
	24–59 months		25 (24.0)
	60 months+		25 (24.0)

Note: SD = standard deviation; IQR = interquartile range; *n* = number; % = percentage; CAD = Canadian Dollars.

**Table 2:** Descriptive characteristics of scaled measures.

Psychological Outcome	Mean (*SD*)	Category	*n* (%)
GAD-7	5.2 (5.1)	Mild	82 (78.9)
		Moderate to Severe	22 (21.2)
PHQ-9	7.0 (5.7)	Mild	77 (74.0)
		Moderate to Severe	27 (26.0)
CWS	13.9 (4.5)	Low	52 (50.0)
		High	52 (50.0)
Distress	3.6 (2.7)	Low	58 (55.6)
		High	46 (44.2)
PTGI	38.9 (26.4)	Low	59 (57.3)
		High	44 (42.7)
**Associate Variables**	**Mean (*SD*)**		
SDI	9.7 (7.4)		
PBQ	19.4 (4.3)		
IPQ	24.6 (8.3)		

Note: *n* = number; % = percentage; GAD-7 = Generalized Anxiety Disorder; PHQ = Patient Health Questionnaire; CWS = Cancer Worry Scale; PTGI = Post-traumatic Growth Inventory; SDI = Social Difficulty Inventory; PBQ = Pandemic-related Behaviour Questionnaire; IPQ = Illness Perception Questionnaire.

**Table 3:** Multivariable logistic regression models.

Characteristic	Statistic	Odds Ratio (95% CI)
Outcome = GAD7		
Active Treatment	Yes vs. No	3.37 (1.05, 10.85)
Social Distress	/unit	1.20 (1.10, 1.32)
Outcome = PHQ9		
Active Treatment	Yes vs. No	3.77 (1.17, 12.16)
Social Distress	/unit	1.25 (1.13, 1.38)
Outcome = CWS		
Employment Status	Working	0.87 (0.30, 2.56)
	Not Working	4.61 (1.23, 17.32)
	Retired	Reference
IPQ	/unit	1.13 (1.06, 1.21)
Outcome = Distress		
Sex	Female vs. Male	6.32 (1.59, 25.06)
Level of Education	High School or Less	1.09 (0.30, 3.99)
	Some Post-Secondary	0.21 (0.06, 0.77)
	University or Postgraduate	Reference
Social Distress	/unit	1.25 (1.14, 1.38)
PBQ	/unit	1.23 (1.08, 1.40)
	Outcome = PTGI	
Age	/year	0.94 (0.91, 0.98)
PBQ	/unit	1.23 (1.09, 1.38)

Note: CI = Confidence interval; GAD-7 = Generalized Anxiety Disorder-7; PHQ9 = Patient Health Questionnaire-9; CWS = Cancer Worry Scale; IPQ = Illness Perception Questionnaire; PBQ = Pandemic-related Behaviour Questionnaire; PTGI = Post-traumatic Growth Inventory.

**Table 4:** Pearson correlation coefficient between psychological outcome variables.

	CWS	PHQ-9	GAD-7	Distress
PTGI	0.24 *	0.10	0.11	0.15
CWS		0.49 **	0.57 **	0.62 **
PHQ-9			0.75 **	0.64 **
GAD-7				0.66 **

* *p* < 0.05; ** *p* < 0.01; Note: PTGI = Post-traumatic Growth Inventory; CWS = Cancer Worry Scale; PHQ = Patient Health Questionnaire; GAD-7 = Generalized Anxiety Disorder.

## Data Availability

The data that support the findings of this study are available from the corresponding author upon request. The data are not publicly available due to privacy and ethical restrictions.

## Supplementary Materials

The following supporting information can be downloaded at: https://www.mdpi.com/article/10.3390/curroncol31090395/s1, Table S1: Univariable Logistic Regression Models.

## References

[1] Chen G., Wu Q., Jiang H., Zhang H., Peng J., Hu J., Chen M., Zhong Y., Xie C. (2020). Fear of disease progression and psychological stress in cancer patients under the outbreak of COVID-19. Psychooncology.

[2] Swainston J., Chapman B., Grunfeld E.A., Derakshan N. (2020). COVID-19 Lockdown and Its Adverse Impact on Psychological Health in Breast Cancer. Front. Psychol..

[3] Gagliardi A.R., Yip C.Y.Y., Irish J., Wright F.C., Rubin B., Ross H., Green R., Abbey S., McAndrews M.P., Stewart D.E. (2021). The psychological burden of waiting for procedures and patient-centred strategies that could support the mental health of wait-listed patients and caregivers during the COVID-19 pandemic: A scoping review. Health Expect..

[4] Han A.Y., Miller J.E., Long J.L., St John M.A. (2020). Time for a Paradigm Shift in Head and Neck Cancer Management During the COVID-19 Pandemic. Otolaryngol. Head Neck Surg..

[5] Miaskowski C., Paul S.M., Snowberg K., Abbott M., Borno H., Chang S., Chen L.M., Cohen B., Hammer M.J., Kenfield S.A. (2020). Stress and Symptom Burden in Oncology Patients during the COVID-19 Pandemic. J. Pain Symptom Manag..

[6] Wang Y., Duan Z., Ma Z., Mao Y., Li X., Wilson A., Qin H., Ou J., Peng K., Zhou F. (2020). Epidemiology of mental health problems among patients with cancer during COVID-19 pandemic. Transl. Psychiatry.

[7] Linden W., Vodermaier A., Mackenzie R., Greig D. (2012). Anxiety and depression after cancer diagnosis: Prevalence rates by cancer type, gender, and age. J. Affect. Disord..

[8] Massicotte V., Ivers H., Savard J. (2021). COVID-19 Pandemic Stressors and Psychological Symptoms in Breast Cancer Patients. Curr. Oncol..

[9] Zomerdijk N., Jongenelis M., Short C.E., Smith A., Turner J., Huntley K. (2021). Prevalence and correlates of psychological distress, unmet supportive care needs, and fear of cancer recurrence among haematological cancer patients during the COVID-19 pandemic. Support. Care Cancer.

[10] Tamiolaki A., Kalaitzaki A.E. (2020). “That which does not kill us, makes us stronger”: COVID-19 and Posttraumatic Growth. Psychiatry Res..

[11] Asmundson G.J.G., Paluszek M.M., Taylor S. (2021). Real versus illusory personal growth in response to COVID-19 pandemic stressors. J. Anxiety Disord..

[12] Calhoun L.G., Tedeschi R.G. (2004). The foundations of posttraumatic growth: New considerations. Psychol. Inq..

[13] Tedeschi R.G., Shakespeare-Finch J., Taku K., Calhoun L.G. (2018). Posttraumatic Growth: Theory, Research, and Applications.

[14] Cordova M.J., Cunningham L.L., Carlson C.R., Andrykowski M.A. (2001). Posttraumatic growth following breast cancer: A controlled comparison study. Health Psychol..

[15] Holland K.D., Holahan C.K. (2003). The relation of social support and coping to positive adaptation to breast cancer. Psychol. Health.

[16] Borrell-Carrió F., Suchman A.L., Epstein R.M. (2004). The biopsychosocial model 25 years later: Principles, practice, and scientific inquiry. Ann. Fam. Med..

[17] Christensen M.M., Friesen M.J., Ponto J.A., Di Tommaso M.J. (2022). Social Isolation Among Individuals with Cancer. Clin. J. Oncol. Nurs..

[18] Zhang A.Y., Koroukian S., Owusu C., Moore S.E., Gairola R. (2023). Socioeconomic correlates of health outcomes and mental health disparity in a sample of cancer patients during the COVID-19 pandemic. J. Clin. Nurs..

[19] Leventhal H., Phillips L.A., Burns E. (2016). The Common-Sense Model of Self-Regulation (CSM): A dynamic framework for understanding illness self-management. J. Behav. Med..

[20] Wierenga K.L., Moore S.E., Pressler S.J., Hacker E.D., Perkins S.M. (2021). Associations between COVID-19 perceptions, anxiety, and depressive symptoms among adults living in the United States. Nurs. Outlook.

[21] Ng D.W.L., Chan F.H.F., Barry T.J., Lam C., Chong C.Y., Kok H.C.S., Liao Q., Fielding R., Lam W.W.T. (2020). Psychological distress during the 2019 Coronavirus Disease (COVID-19) pandemic among cancer survivors and healthy controls. Psychooncology.

[22] Jammu A.S., Chasen M.R., Lofters A.K., Bhargava R. (2021). Systematic rapid living review of the impact of the COVID-19 pandemic on cancer survivors: Update to August 27, 2020. Support. Care Cancer.

[23] Veeraiah S., Chidambaram S., Sudhakar R., Ganesharaja S., Krishnamurthy A., Dhanushkodi M., Swaminathan R. (2022). Psychosocial issues and concerns of cancer patients due to COVID-19 pandemic lockdown. PLoS Glob. Public Health.

[24] Chapman B., Swainston J., Grunfeld E.A., Derakshan N. (2020). COVID-19 Outbreak Effects on Job Security and Emotional Functioning Amongst Women Living with Breast Cancer. Front. Psychol..

[25] Bafunno D., Romito F., Lagattolla F., Delvino V.A., Minoia C., Loseto G., Dellino M., Guarini A., Catino A., Montrone M. (2021). Psychological well-being in cancer outpatients during COVID-19. J. BUON.

[26] Zhang H., Yin J., Wang X., Yuan D., Zhu K., Li K., Xu G., Dang C., Jia R., Zhang Y. (2021). Patients’ Responses to the Sudden Interruption of Chemotherapy during the Outbreak of the Novel Coronavirus: A Cross-Sectional Study. Cancer Manag. Res..

[27] Obispo-Portero B., Cruz-Castellanos P., Jiménez-Fonseca P., Rogado J., Hernandez R., Castillo Trujillo O.A., Asensio-Martínez E., González-Moya M., Carmona-Bayonas A., Calderon C. (2022). Anxiety and depression in patients with advanced cancer during the COVID-19 pandemic. Support. Care Cancer.

[28] Wright P., Smith A.B., Keding A., Velikova G. (2011). The Social Difficulties Inventory (SDI): Development of subscales and scoring guidance for staff. Psychooncology.

[29] Broadbent E., Petrie K.J., Main J., Weinman J. (2006). The brief illness perception questionnaire. J. Psychosom. Res..

[30] Karademas E.C., Bati A., Karkania V., Georgiou V., Sofokleous S. (2013). The association between pandemic influenza A (H1N1) public perceptions and reactions: A prospective study. J. Health Psychol..

[31] Kroenke K., Spitzer R.L., Williams J.B. (2001). The PHQ-9: Validity of a brief depression severity measure. J. Gen. Intern. Med..

[32] Spitzer R.L., Kroenke K., Williams J.B., Löwe B. (2006). A brief measure for assessing generalized anxiety disorder: The GAD-7. Arch. Intern. Med..

[33] Custers J.A., van den Berg S.W., van Laarhoven H.W., Bleiker E.M., Gielissen M.F., Prins J.B. (2014). The Cancer Worry Scale: Detecting fear of recurrence in breast cancer survivors. Cancer Nurs..

[34] Cutillo A., O’Hea E., Person S., Lessard D., Harralson T., Boudreaux E. (2017). The Distress Thermometer: Cutoff Points and Clinical Use. Oncol. Nurs. Forum.

[35] Tedeschi R.G., Calhoun L.G. (1996). The Posttraumatic Growth Inventory: Measuring the positive legacy of trauma. J. Trauma. Stress.

[36] Mazor Y., Gelkopf M., Mueser K.T., Roe D. (2016). Posttraumatic Growth in Psychosis. Front. Psychiatry.

[37] Wu X., Kaminga A.C., Dai W., Deng J., Wang Z., Pan X., Liu A. (2019). The prevalence of moderate-to-high posttraumatic growth: A systematic review and meta-analysis. J. Affect. Disord..

[38] Cormio C., Muzzatti B., Romito F., Mattioli V., Annunziata M.A. (2017). Posttraumatic growth and cancer: A study 5 years after treatment end. Support. Care Cancer.

[39] Helgeson V.S., Reynolds K.A., Tomich P.L. (2006). A meta-analytic review of benefit finding and growth. J. Consult. Clin. Psychol..

[40] Boerner M., Joseph S., Murphy D. (2020). A Theory on Reports of Constructive (Real) and Illusory Posttraumatic Growth. J. Humanist. Psychol..

[41] Lafuenti L., Dinapoli L., Mastrilli L., Savoia V., Linardos M., Masetti R., Tortora G., Valentini V., Scambia G., Chieffo D.P.R. (2023). Post-traumatic growth in oncological patients during the COVID-19 pandemic. Health Psychol. Rep..

[42] Calhoun L.G., Tedeschi R.G., Calhoun L.G., Tedeschi R.G. (2006). Chapter 1: The Foundations of Posttraumatic Growth: An Expanded Framework. Handbook of Posttraumatic Growth: Research & Practice.

[43] Howden K., Glidden C., Romanescu R.G., Hatala A., Scott I., Deleemans J., Chalifour K., Eaton G., Gupta A.A., Bolton J.M. (2021). A Cross-Sectional Survey Exploring the Impact of the COVID-19 Pandemic on the Cancer Care of Adolescents and Young Adults. Curr. Oncol..

[44] Tan H.J., Marks L.S., Hoyt M.A., Kwan L., Filson C.P., Macairan M., Lieu P., Litwin M.S., Stanton A.L. (2016). The Relationship between Intolerance of Uncertainty and Anxiety in Men on Active Surveillance for Prostate Cancer. J. Urol..

[45] Buhr K., Dugas M.J. (2009). The role of fear of anxiety and intolerance of uncertainty in worry: An experimental manipulation. Behav. Res. Ther..

[46] Caycho-Rodríguez T., Tomás J.M., Vilca L.W., Carbajal-León C., Cervigni M., Gallegos M., Martino P., Barés I., Calandra M., Anacona C.A.R. (2021). Socio-Demographic Variables, Fear of COVID-19, Anxiety, and Depression: Prevalence, Relationships and Explanatory Model in the General Population of Seven Latin American Countries. Front. Psychol..

[47] Lotfi-Kashani F., Vaziri S., Akbari M.E., Kazemi-Zanjani N., Shamkoeyan L. (2014). Predicting Post Traumatic Growth Based upon Self-Efficacy and Perceived Social Support in Cancer Patients. Iran. J. Cancer Prev..

[48] Ashley L., Keding A., Brown J., Velikova G., Wright P. (2013). Score equivalence of electronic and paper versions of the Social Difficulties Inventory (SDI-21): A randomised crossover trial in cancer patients. Qual. Life Res..

[49] Momenimovahed Z., Salehiniya H., Hadavandsiri F., Allahqoli L., Günther V., Alkatout I. (2021). Psychological Distress Among Cancer Patients During COVID-19 Pandemic in the World: A Systematic Review. Front. Psychol..

[50] Trevino K.M., Fasciano K., Prigerson H.G. (2013). Patient-oncologist alliance, psychosocial well-being, and treatment adherence among young adults with advanced cancer. J. Clin. Oncol..

