# Supplementary material for: Biopsychosocial Associates of Psychological Distress and Post-Traumatic Growth among Canadian Cancer Patients during the COVID-19 Pandemic
Source: Curr Oncol. 2024 Sep 10;31(9):5354–66. doi: 10.3390/curroncol31090395 (PMC11431811; doi:10.3390/curroncol31090395)
Supplement: Supplementary file 1 [file curroncol-31-00395-s001.zip › curroncol-3156828-supplementary.pdf]

Table S1: Univariable Logistic Regression Models

**Characteristic**	**Statistic**	**Outcome = GAD**	**Outcome=PHQ**	**Outcome=FCR**	**Outcome=Distress**	**Outcome = PTGI**
Age	/ year	0.99 (0.96, 1.03)	0.99 (0.96, 1.03)	**0.97 (0.94, 1.00)**	**0.95 (0.91, 0.98)**	**0.95 (0.91, 0.98)**
Sex	Female vs Male	1.37 (0.41, 4.55)	1.38 (0.46, 4.15)	1.89 (0.74, 4.83)	**2.93 (1.05, 8.14)**	2.71 (0.97, 7.55)
Relationship Status	Married/Common-law vs single	0.75 (0.27, 2.09)	**0.34 (0.13, 0.86)**	0.41 (0.17, 1.01)	0.62 (0.26, 1.46)	0.71 (0.30, 1.68)
Employment Status	Working<br>Not Working<br>Retired	1.77 (0.54, 5.85)<br>4.21 (1.23, 14.37)<br>Reference	1.52 (0.53, 4.36)<br>2.60 (0.83, 8.14)<br>Reference	**1.77 (0.72, 4.33)**<br>**7.50 (2.17, 25.94)**<br>**Reference**	**4.17 (1.62, 10.74)**<br>**3.89 (1.31, 11.51)**<br>**Reference**	2.65 (1.06, 6.65)<br>2.59 (0.90, 7.48)<br>Reference
Level of Education	High School or Less<br>Some Post-Secondary<br>University or Postgraduate	2.33 (0.63, 8.70)<br>2.59 (0.79, 8.48)<br>Reference	0.79 (0.23, 2.67)<br>1.27 (0.47, 3.47)<br>Reference	0.91 (0.33, 2.49)<br>0.86 (0.35, 2.10)<br>Reference	0.94 (0.34, 2.58)<br>0.67 (0.27, 1.67)<br>Reference	1.07 (0.38, 3.00)<br>1.28 (0.52, 3.15)<br>Reference
Ethnicity	White vs other	1.08 (0.21, 5.50)	**0.19 (0.05, 0.74)**	0.39 (0.10, 1.62)	0.49 (0.13, 1.87)	0.46 (0.12, 1.74)
Household Income	<$60,000<br>$60,000 to $99,999<br>$100,000 or more<br>Missing	4.50 (0.88, 23.04)<br>4.94 (0.91, 26.77)<br>Reference<br>2.82 (0.46, 17.21)	7.80 (1.57, 38.73)<br>4.94 (0.91, 26.77)<br>Reference<br>3.75 (0.65, 21.74)	2.10 (0.74, 5.97)<br>1.15 (0.38, 3.53)<br>Reference<br>1.24 (0.39, 3.94)	1.28 (0.46, 3.61)<br>0.97 (0.32, 3.00)<br>Reference<br>1.02 (0.32, 3.27)	0.44 (0.15, 1.27)<br>1.18 (0.39, 3.59)<br>Reference<br>0.67 (0.21, 2.17)
People in Household	/ person	0.88 (0.61, 1.26)	0.66 (0.42, 1.04)	0.95 (0.79, 1.15)	0.83 (0.62, 1.13)	0.97 (0.80, 1.17)
Time Since Diagnosis	/ year	1.01 (0.97, 1.04)	0.99 (0.95, 1.04)	0.99 (0.95, 1.03)	0.99 (0.95, 1.03)	0.93 (0.85, 1.02)
	<1 year<br>12-23 months<br>24-59 months<br>60 months+	Reference<br>1.00 (0.22, 4.49)<br>2.71 (0.70, 10.48)<br>1.82 (0.45, 7.39)	Reference<br>0.46 (0.13, 1.60)<br>0.78 (0.24, 2.54)<br>0.63 (0.19, 2.13)	Reference<br>1.58 (0.53, 4.68)<br>2.55 (0.83, 7.80)<br>2.16 (0.71, 6.57)	Reference<br>1.83 (0.62, 5.43)<br>1.13 (0.37, 3.47)<br>1.57 (0.52, 4.75)	Reference<br>1.47 (0.50, 4.34)<br>0.51 (0.16, 1.57)<br>0.51 (0.16, 1.57)
Treatment Intent	Curative<br>Palliative<br>Control/Unknown	1.02 (0.38, 2.77)<br>Reference<br>0.52 (0.06, 4.85)	1.47 (0.56, 3.85)<br>Reference<br>1.21 (0.20, 7.18)	1.02 (0.45, 2.32)<br>Reference<br>1.76 (0.37, 8.46)	1.79 (0.77, 4.16)<br>Reference<br>1.85 (0.40, 8.62)	**2.71 (1.13, 6.50)**<br>**Reference**<br>**0.79 (0.14, 4.53)**
Comorbidities	/ comorbidity	1.13 (0.92, 1.38)	1.16 (0.96, 1.41)	1.14 (0.95, 1.36)	1.04 (0.87, 1.24)	1.04 (0.87, 1.24)
Active Treatment	Yes vs No	**2.86 (1.08, 7.55)**	**3.47 (1.38, 8.72)**	**2.46 (1.04, 5.86)**	**2.89 (1.22, 6.84)**	1.13 (0.48, 2.63)

Disease Site	Breast<br>Genitourinary<br>Hematology<br>Other	0.92 (0.22, 3.86)<br>1.47 (0.30, 7.19)<br>0.61 (0.09, 4.37)<br>Reference	0.85 (0.23, 3.16)<br>0.78 (0.17, 3.62)<br>1.00 (0.19, 5.15)<br>Reference	0.63 (0.19, 2.04)<br>0.68 (0.18, 2.66)<br>1.35 (0.30, 6.18)<br>Reference	0.72 (0.22, 2.36)<br>0.23 (0.05, 1.01)<br>0.56 (0.13, 2.51)<br>Reference	1.80 (0.53, 6.08)<br>0.42 (0.09, 1.98)<br>2.40 (0.52, 10.99)<br>Reference
SDI - Total	/ unit	**1.20 (1.10, 1.31)**	**1.26 (1.15, 1.39)**	**1.13 (1.06, 1.21)**	**1.18 (1.09, 1.27)**	1.02 (0.97, 1.08)
SDI-Everyday	/ unit	**1.21 (1.06, 1.38)**	**1.31 (1.14, 1.50)**	**1.14 (1.01, 1.28)**	**1.17 (1.04, 1.31)**	1.03 (0.92, 1.15)
SDI-money	/ unit	**1.64 (1.28, 2.09)**	**1.55 (1.23, 1.95)**	**1.39 (1.10, 1.74)**	**2.02 (1.42, 2.86)**	0.99 (0.84, 1.16)
SDI-other	/ unit	**1.50 (1.23, 1.83)**	**1.71 (1.36, 2.14)**	**1.43 (1.19, 1.70)**	**1.49 (1.24, 1.80)**	1.12 (0.98, 1.28)
IPQ	/ unit	**1.09 (1.03, 1.16)**	**1.11 (1.04, 1.18)**	**1.13 (1.07, 1.20)**	**1.12 (1.05, 1.18)**	**1.05 (1.00, 1.11)**
PBQ	/ unit	1.10 (0.99, 1.23)	1.04 (0.93, 1.15)	1.07 (0.97, 1.17)	1.10 (1.00, 1.21)	**1.22 (1.09, 1.36)**

Note: GAD-7 = Generalized Anxiety Disorder; PHQ = Patient Health Questionnaire; PTGI: Posttraumatic Growth Inventory; CWS = Cancer Worry Scale; SDI = Social Difficulty Inventory; PBQ = Pandemic-related Behaviour Questionnaire; IPQ = Illness Perception Questionnaire
